# Simultaneous Ultrasound-Assisted Hybrid Polyzwitterion/Antimicrobial Peptide Nanoparticles Synthesis and Deposition on Silicone Urinary Catheters for Prevention of Biofilm-Associated Infections

**DOI:** 10.3390/nano11113143

**Published:** 2021-11-21

**Authors:** Aleksandra Ivanova, Kristina Ivanova, Tzanko Tzanov

**Affiliations:** Grup de Biotecnologia Molecular i Industrial, Department of Chemical Engineering, Universitat Politècnica de Catalunya, Rambla Sant Nebridi 22, 08222 Terrassa, Spain; aleksandra.asenova@upc.edu (A.I.); kristina.ivanova@upc.edu (K.I.)

**Keywords:** Polymyxin B, poly(sulfobetaine) methacrylate, sonochemistry, nanohybrids, antibacterial activity, biofilm inhibition, catheter-associated bacterial infections

## Abstract

Nosocomial infections caused by antibiotic-resistant bacteria are constantly growing healthcare threats, as they are the reason for the increased mortality, morbidity, and considerable financial burden due to the poor infection outcomes. Indwelling medical devices, such as urinary catheters, are frequently colonized by bacteria in the form of biofilms that cause dysfunction of the device and severe chronic infections. The current treatment strategies of such device-associated infections are impaired by the resistant pathogens but also by a risk of prompting the appearance of new antibiotic-resistant bacterial mechanisms. Herein, the one-step sonochemical synthesis of hybrid poly(sulfobetaine) methacrylate/Polymyxin B nanoparticles (pSBMA@PM NPs) coating was employed to engineer novel nanoenabled silicone catheters with improved antifouling, antibacterial, and antibiofilm efficiencies. The synergistic mode of action of nanohybridized zwitterionic polymer and antimicrobial peptide led to complete inhibition of the nonspecific protein adsorption and up to 97% reduction in *Pseudomonas aeruginosa* biofilm formation, in comparison with the pristine silicone. Additionally, the bactericidal activity in the hybrid coating reduced the free-floating and surface-attached bacterial growth by 8 logs, minimizing the probability for further *P. aeruginosa* spreading and host invasion. This coating was stable for up to 7 days under conditions simulating the real scenario of catheter usage and inhibited by 80% *P. aeruginosa* biofilms. For the same time of use, the pSBMA@PM NPs coating did not affect the metabolic activity and morphology of mammalian cells, demonstrating their capacity to control antibiotic-resistant biofilm-associated bacterial infections.

## 1. Introduction

The use of implantable medical devices such as indwelling urinary catheters is imperative in the field of modern medicine for managing urinary retention and incontinence in hospitalized patients [1]. Urinary catheters are a predisposing factor for bacterial colonization and the formation of antibiotic-resistant biofilms, causing difficult-to-treat, healthcare-associated infections (HAI) and prolonged hospitalization [2]. Bacterial biofilms on medical devices have serious negative impacts on human health since they cause severe chronic infections and lead to increased mortality, morbidity, and a considerable financial burden to the healthcare systems [3,4]. Catheter-associated urinary tract infections (CAUTIs) are among the most common HAIs and represent 80% of the urinary tract infections currently treated in hospitals [5].

The high incidence of CAUTIs is associated with frequent replacement of the device and aggressive antibiotic therapy [6], resulting in patients’ discomfort and selection for drug-resistant bacterial species [7]. Although silicone catheters were introduced into the clinical practice as a more efficient alternative to the latex materials, the rapid formation of the so-called conditioning layer composed of nonspecifically attached proteins that serve as anchoring spots for establishing resistance to the host defenses, and conventional antibiotic biofilm structure limits their long-term utilization [8]. Catheters’ functionalization with conventional antibiotics or metal/metal oxide nanoparticles (NPs) has been suggested as a prominent measure to decrease the prevalence of biofilm diseases [9,10,11]. However, these antimicrobial agents may induce side effects such as inflammation and cytotoxicity, and instigate the appearance of resistance [11,12]. In consequence, significant efforts are being made to design novel and more efficient antibiofilm surfaces based on antifouling polymers, biopolymers [13], or anti-infective/bactericidal enzymes [14,15,16] with low potential for promoting resistance occurrence. In our group, we developed hybrid multilayer coatings of quorum quenching acylase and matrix-degrading amylase on urinary catheters able to inhibit the biofilm formation in vitro and in vivo [14,17]. We also reported a two-step biotechnology-based approach for the covalent functionalization of silicone catheters with antifouling zwitterionic coating using enzyme laccase. Urinary catheters were first plasma activated and prelaminated to allow the laccase-assisted grafting of the natural phenolic compound gallic acid (GA). Subsequently, the tethered GA residues were activated by laccase to phenoxy radicals, triggering an enzymatically initiated radical polymerization of zwitterionic sulfobetaine methacrylate monomers on the silicone catheters [13]. Although these strategies are effective in diminishing the biofilm establishment on the surface, they do not aim to kill the pathogens, and the threat of spreading and infection occurrence may still persist.

This work extends beyond the described state of the art by developing a novel bifunctional nanoenabled coating for simultaneous inhibition of nonspecific proteins adherence and initial bacterial settlement on silicone surface, together with the effective killing of the incoming *P. aeruginosa* bacteria upon contact. Contrary to the most frequently used surface functionalization techniques, which are time consuming and environmentally hazardous [18], we employed a single-step green sonochemical approach for simultaneous synthesis of hybrid zwitterion/peptide NPs and their deposition on catheters, without the need for any surface pretreatment or coating additives. Ultrasound nanoenabled coating technology is water based, implies low temperature (20 °C), and allows the generation of durable “ready-to-use” surface nanostructured antimicrobial products [19]. Furthermore, the nanosized combination of bactericidal Polymyxin B and antifouling poly(sulfobetaine) methacrylate (pSBMA) is envisaged to improve the peptide stability [18], repelling the proteins and residues of dead bacterial cells, and synergistically enhance the antibiofilm activity of the coatings at lower nontoxic concentrations of the actives. Nanoformulation of biocides endows them with additional mechanisms of antimicrobial action, e.g., membrane disruption and “invisibility” to the defense system of bacteria that does not recognize them as a threat. The improved physical interaction and disturbance of the bacterial membranes by these nanoentities will lead to bacteria eradication, avoiding resistance development.

The antibacterial activity of the developed hybrid nanocomposites coating was assessed against *Pseudomonas aeruginosa* as the most common pathogen causing indwelling medical devices-associated infections. Furthermore, the antibiofilm efficiency of such NPs coating was assessed in vitro in both static and dynamic conditions, under constant artificial urine flow mimicking the real situation during catheterization. The biocompatibility was also assessed by monitoring the changes in the mammalian cell lines’ metabolic activities and morphology upon exposure to the coatings for 1 and 7 days.

## 2. Materials and Methods

### 2.1. Materials

Polymyxin B and [2-(methacryloyloxy)ethyl]dimethyl-(3-sulfopropyl)ammonium hydroxide (sulfobetaine methacrylate, SBMA) (95%) were purchased from Sigma-Aldrich (Madrid, Spain). Potassium persulfate (K_2_S_2_O_8_, ≥99%) was purchased from Chem-Lab (Zedelgem, Belgium). The pSBMA was prepared as previously described [20]. Briefly, 0.1 mol.% of initiator K_2_S_2_O_8_ was added to 0.2 M of an aqueous solution of SBMA monomer. The polymerization reaction proceeded at 60 °C for 6 h under continuous stirring. Subsequently, the polymer solution was transferred to dialysis membranes (cut off 13 kDa) against distilled water for 1 week. Then, the obtained solution was lyophilized for 2 days and stored at room temperature until further use.

Gram-negative *Pseudomonas aeruginosa* (*P. aeruginosa*, ATCC 10145), human fibroblast (ATCC-CRL-4001, BJ-5ta), and keratinocyte (HaCaT cell line) cells were obtained from the American Type Culture Collection (ATCC LGC Standards, Barcelona, Spain). Live/Dead^®^ Kits for mammalian cells viability kit was provided by Thermo Fischer Scientific (Sant Cugat del Vallès, Spain). AlamarBlue Cell Viability Reagent and Live/Dead BacLight Kit (Molecular probes L7012) were obtained from Invitrogen, Life Technologies Corporation (Madrid, Spain). Polydimethyl/vinylmethyl siloxane urinary (Foley) catheters and silicone sheets designated according to ASTM D 1418 were purchased by Degania Silicone Ltd. (Degania Bet, Israel). All other chemicals were provided from Sigma-Aldrich (Madrid, Spain) and used without further purification.

### 2.2. Ultrasound Coating of Silicone Materials with Polymyxin B and pSBMA NPs

The sonochemical coating was carried out using an ultrasonic transducer Ti-horn (20 kHz, VC750, Sonics & Materials, Inc., Newtown, CT, USA). The silicone strips (1 × 1 cm^2^) were immersed in the ultrasonic pot containing 100 mL aqueous solution of Polymyxin B (1 mg/mL) and pSBMA (0.5 mg/mL), and the coating of the silicone samples was carried out for 15 min at 20 °C, amplitude of 50%, and energy supply 29.5 W/cm^2^. Silicones, coated with 1 mg/mL Polymyxin B and 0.5 mg/mL pSBMA, were used as control samples. The ultrasonic horn was dipped 2 cm in the solution at a distance from the silicone of approximately 5 cm. Thereafter, the samples were thoroughly washed with distilled water. For the tests under dynamic conditions, whole-size silicone 18 fr Foley catheter was coated as described above.

### 2.3. Surface-Morphology and Surface-Wetting Characterization

The morphology of the 1 × 1 cm^2^ pristine silicone and hybrid coated silicone was investigated by a field emission scanning electron microscope (SEM, Quanta 650 FEG, Field Electron and Ion Company, Hillsboro, OR, USA) at vacuum mode, operating at 20 kV at 2500× magnification. Contact angle measurements were carried out with mQ water to determine the hydrophobicity and surface properties of the hybrid nanocomposites. Briefly, the measurements were performed at room temperature in the air using DSA25 Drop Shape Analyzer Krüss Instruments (Instrumentación analítica S.A., Prat del Llobregat, Spain) and applying the sessile drop method. Liquid drops of 2 µL were applied to the material surface. The contact angle measurements for the determination of the hydrophilic/hydrophobic characteristics were estimated by the two-dimensional projection of the droplet on both left and right sides.

### 2.4. Protein Adsorption Test

Silicone samples (1 cm × 1 cm) were immersed in a 1 mg mL^−1^ albumin-fluorescein isothiocyanate conjugate (FITC–BSA) solution (*w*/*v*) for 30 min to simulate the immediate process of protein attachment preceding the biofilm formation. After that, the samples were washed with distilled water and dried with nitrogen. The protein attachment on the pristine and coated silicone surfaces was evaluated using a fluorescence microscope NIKON Eclipse Ti-S (Nikon Instruments, Inc., Amstelveen, The Netherlands).

### 2.5. Antibacterial Activity of pSBMA@PM Coated Catheters

The antibacterial activity of the functionalized silicone materials was assessed against Gram-negative *P. aeruginosa*. Briefly, single colonies of bacteria were grown in Mueller Hinton Broth at 37 °C and 230 rpm overnight. Then, the bacteria were diluted in 100 mM phosphate-buffered saline (PBS, pH 6.8) (colony forming units (CFU) mL^−1^ ~10^8^); subsequently, 1 piece (1 × 1 cm^2^) of silicone was placed with 1.5 mL of bacteria in 15 mL sterile falcons and incubated for 24 h at 37 °C and 230 rpm shaking. The bacterial withdrawn suspensions were serially diluted in sterile 100 mM PBS, plated on selective Cetrimide agar, and incubated for 24 h for 37 °C, and the viable bacteria were counted using the drop count method.

### 2.6. Biofilm Inhibition Properties

#### 2.6.1. Crystal Violet Assay

The ability of the developed coatings to impede the biofilm formation on the modified silicone materials was assessed with *P. aeruginosa* as previously described [14]. Briefly, 1 × 1 cm^2^ of silicone was incubated with 1.5 mL of bacterium (OD_600_ = 0.01) in tryptic soy broth (TSB) in a 24-well microplate. The microplate was incubated for 24 h at 37 °C under static conditions, allowing the bacteria to colonize the silicone materials and form biofilms. Following the incubation, the nonadhered bacterial cells were washed three times with 2 mL of sterile 100 mM PBS (pH 6.8), and the bacterial biofilms were fixed for 60 min at 60 °C and stained with 1 mL of 0.1% (*w*/*v*) crystal violet solution for 15 min. Subsequently, the silicone pieces were placed with 1 mL of 30% acetic acid (*v*/*v*) to redissolve the crystal violet dye fixed on the samples. The absorbance of the resulting solutions was measured at 595 nm.

#### 2.6.2. Cell Viability Assay

*P. aeruginosa* were allowed to grow and form biofilm onto the silicone pieces for 24 h, and the nonadhered cells were washed three times with sterile 100 mM PSB (pH 6.8). The silicone pieces were placed in sterile 15 mL falcons and 2 mL PBS and sonicated in a US bath at 37 °C for 15 min in terms to remove the biofilm of the silicone surface. Further, the bacterial cells of the formed biofilm were plated on selective Cetrimide agar, the plates were incubated for 24 h for 37 °C, and the survived bacteria were counted using the drop count method.

#### 2.6.3. Live/Dead Kit Assay

The live and dead bacteria in the biofilms formed on the silicones were also assessed using Live/Dead BacLight Kit assay. After biofilm development, the samples were stained with a mixture of Syto 9 and Propidium iodide (PI) (1:1) for 15 min. The biofilms were rinsed with 100 µL of 100 mM PBS (pH 6.8). Subsequently, the samples were analyzed by fluorescent microscopy at Ex/Em 480/500 for Syto 9 staining the live bacteria and PI at Ex/Em 490/635 labeling the dead bacteria. The stained biofilms were observed under 20× magnification. The live cells were stained in green and the dead ones in red.

Fluorescence microscopy at Ex/Em (480/500) for Syto^®^ 9 labeling the nucleic acid of all bacteria, with intact and damaged membranes in green and at Ex/Em (490/635) for propidium iodide quenching the green fluorescence of Syto^®^ 9 dye after penetration into the damaged cells, consequently staining dead bacteria in red.

### 2.7. Dynamic Biofilm Inhibition Tests

The ability of the hybrid pSBMA@PM coating to impair the biofilm formation was evaluated under dynamic conditions using an in vitro physical model of the catheterized human bladder [14]. Briefly, the nontreated and pSBMA@PM NPs-coated silicone Foley catheters (18 fr) were inserted into the sterile model and the catheter’s balloon was inflated with 5 mL 100 mM PBS (pH 6.8). Subsequently, the bladder was filled up to the catheter’s eye with sterile artificial urine, pH 6.8, prepared according to UNE EN1616 (Sterile Urethral Catheters for Single Use), and supplemented with 1 mg mL^−1^ TSB Gram-negative *P. aeruginosa* (final OD_600_ = 0.01). The model was maintained at 37 °C for 7 days and supplied with sterile artificial urine at a flow rate of 1 mL min^−1^. Then, the catheter was removed, and the total biofilm mass formed on the surface (catheter’s tip and balloon) was studied using crystal violet assay. Nontreated silicone Foley catheter served as a control sample (no biofilm inhibition).

### 2.8. Biocompatibility Assessment

The cytotoxicity of the coated catheters was evaluated using human fibroblast cells (BJ-5ta) and keratinocytes (HaCaT). The functionalized catheters were placed in contact with the previously cultured cells, and the viable cells were quantified after 24 h and 7 days of contact using AlamarBlue Assay Kit (AlamarBlue^®^, Invitrogen, Sant Cugat del Vallès, Spain) [21]. The cells’ morphology was also observed by Live/Dead^®^ Viability/Cytotoxicity Assay Kit (Thermo Fisher Scientific, Sant Cugat del Vallès, Spain) for mammalian cells after exposure of the cells to the coated catheters for 24 h and 7 days as previously described [22].

## 3. Results and Discussion

### 3.1. Silicone Functionalization with Hybrid pSBMA@PM NPs

**Figure 1:**
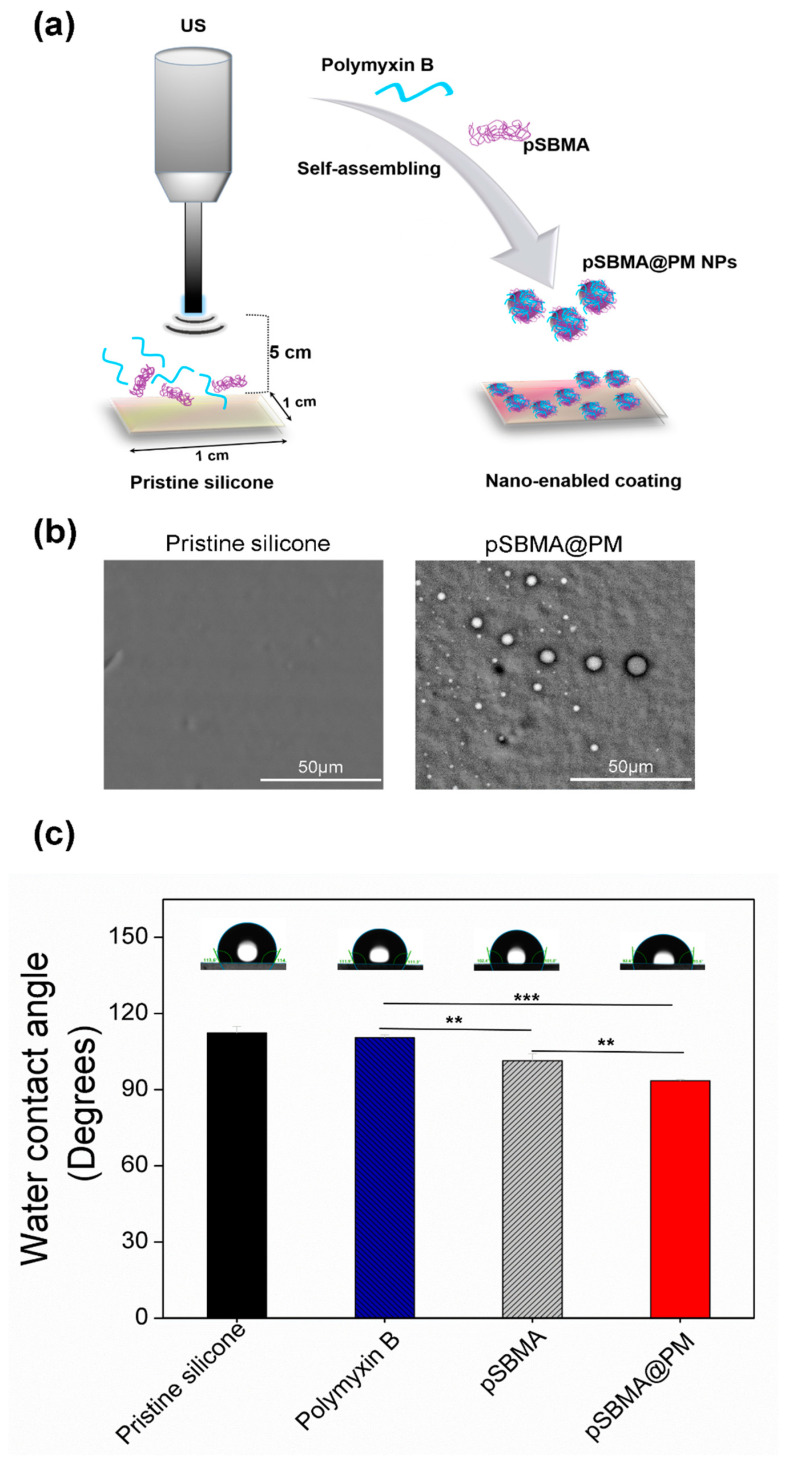
(**a**) Schematic representation of US-assisted pSBMA@PM NPs formulation and their deposition onto silicone surface. Surface characterization of sonochemically coated with pSBMA and Polymyxin B silicone materials: (**b**) SEM images of pristine and pSBMA@PM coated silicone catheter and (**c**) Water contact angles measurements of the developed nanocoatings. All data are mean values of three independent experiments. Stars represent the statistical differences between the different groups of samples, ** *p* < 0.01; *** *p* < 0.001.

The high-intensity US, supported by electrostatically driven self-assembly, was employed to develop in situ assembled dual active hybrid NPs of pSBMA and Polymyxin B and simultaneously deposit them on silicone. The process for US-assisted NP production and coating on silicone materials is schematically illustrated in Figure 1a. Polymyxin B is a cationic peptide with positively charged amino residues that bind with the anionic sites on the zwitterionic polymer, leading to successful NPs formulation. Simultaneously, the resulting hybrid pSBMA@PM NPs were deposited onto silicon surfaces in a “throwing stone” mode under the US field. The surface morphology of the developed hybrid pSBMA@PM coating was investigated using SEM. The obtained SEM images (Figure 1b) revealed successful silicone functionalization with spherical and uniform-sized nanohybrids. A larger population of spheres of 3 µm, however, were also observed, which is a common phenomenon associated with the electrostatic-driven self-assembling complexation of small peptides with zwitterionic polymers [23]. The coating deposition was further confirmed by the changes in the wettability of the coated silicone (Figure 1c). The pristine silicone is hydrophobic with a water contact angle of 112.3° ± 2.5°. Upon coating with pSBMA and Polymyxin B, the water contact angle decreased statistically significantly (Figure 1c) to 93.6° ± 0.4°, whereas the values obtained for the silicone functionalized with pSBMA or Polymyxin B were 101.4° ± 2.7° and 110.5° ± 1.1°, respectively. Apparently, the zwitterionic polymer improved the hydrophilicity of the surface, and it is expected to reduce the nonspecific protein attachment and biofilm growth on catheters [13].

### 3.2. Initial Protein Attachment

**Figure 2:**
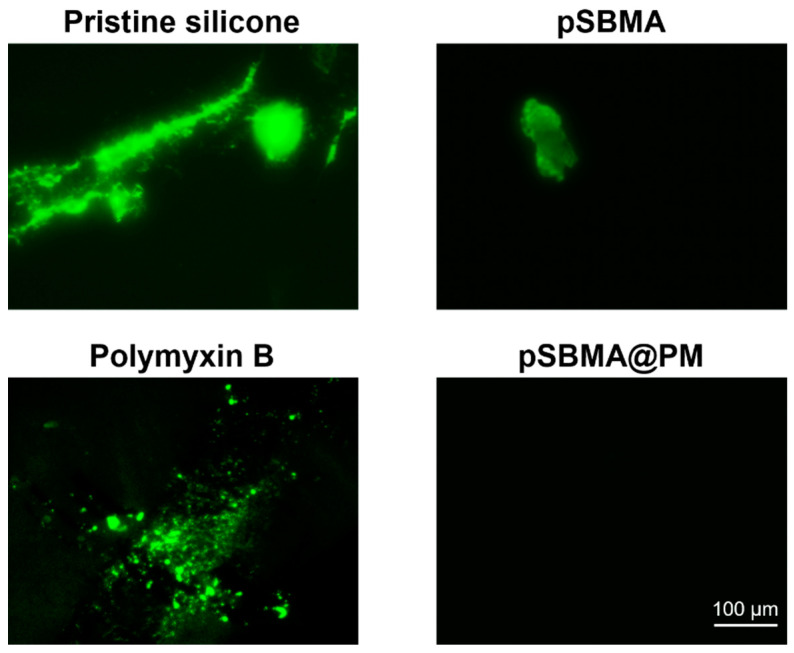
Fluorescence images taken after 30 min of incubation in 1 mg mL^−^^1^ of FITC-labeled BSA solution.

The nonspecifically attached proteins on the surface of the hydrophobic catheters are believed to trigger the initial bacterial adherence and biofilm formation [24]. The protein adsorption on the pristine and coated silicone samples was evaluated by fluorescence microscopy using FITC labeled BSA protein. Figure 2 shows the FITC-labeled protein attached to the pristine silicone, Polymyxin B, pSBMA, and hybrid silicones. Untreated and Polymyxin B treated silicones strongly adsorbed BSA, forming aggregates that would further serve as anchoring points for bacterial cells to adhere to the surface and initiate biofilm growth. As expected, pSBMA functionalized silicone reduced the protein adsorption, and this is in agreement with the results from the biofilm inhibition tests obtained further. BSA did not adhere to the surface of the zwitterion (pSBMA)/Polymyxin B modified silicone surface, confirming their antifouling potential. The antifouling pSBMA is able to inhibit the conditioning layer formation and impair the bacterial colonization onto silicone surface due to the formation of hydration film, disrupting the hydrophobic interactions between the silicone material and the proteins.

### 3.3. Antibacterial Activity of the Hybrid pSBMA@PM NP Coating

The antibacterial activity of the hybrid nanocoating was evaluated against *P. aeruginosa*, which is one of the most common Gram-negative pathogens, associated with foreign body-related infections. Polymyxin B is a cationic polypeptide that is highly efficient against Gram-negative bacteria due to the enhanced interaction with the lipopolysaccharides in their outer membrane, leading to cell membrane disruption and death [25,26]. Such bactericidal mechanisms of action do not exert selective pressure and, therefore, reduce the probability of the appearance of resistance [25].

**Figure 3:**
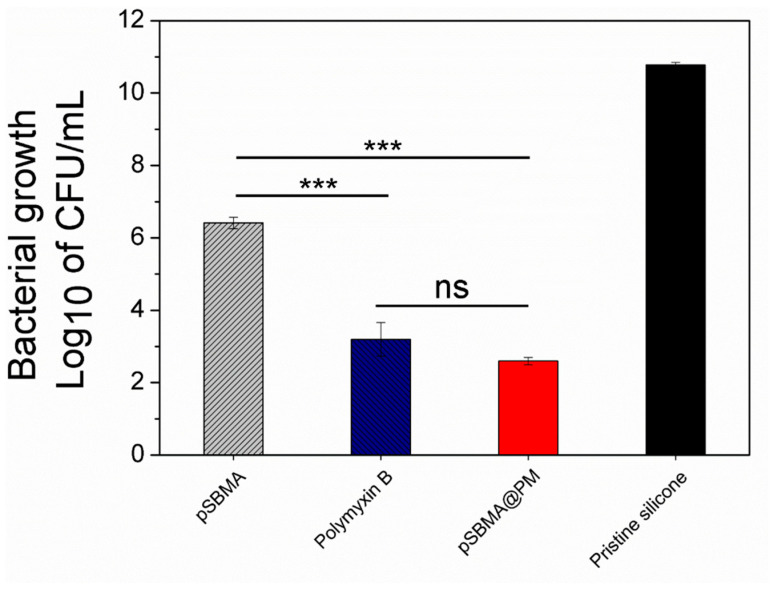
Antibacterial activity of the coated silicones against *P. aeruginosa*. All data are mean values of three independent experiments. Stars represent the statistical differences between the different groups of samples (*** *p* < 0.001); ns—not significantly different (*p* > 0.05).

Catheters coated only with the peptide demonstrated up to seven-log reduction in *P. aeruginosa* free-floating bacterial growth (Figure 3). In the same fashion, hybrid pSBMA@PM coating led to similar bacterial growth reduction due to the Polymyxin B-induced bacterial death. Although zwitterionic polymers are mainly used to inhibit initial protein and bacterial adhesion [8,13], the silicones functionalized with pSBMA unexpectedly reduced *P. aeruginosa* viability by four logs. The US-assisted nanotransformation of pSBMA probably led to changes in the polymer conformation and surface charge orientations of the zwitterionic ligands imparting antimicrobial functionalities due to the enhanced interaction with the bacterial cell membrane [27].

### 3.4. Inhibition of P. aeruginosa Biofilm Formation by the Hybrid pSBMA@PM NP Coating

**Figure 4:**
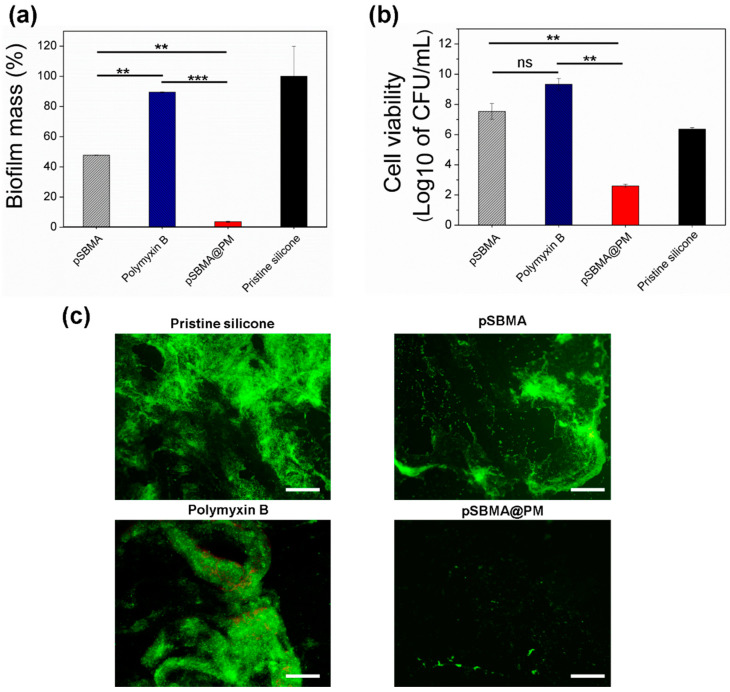
Inhibition of *P. aeruginosa* biofilm formation by untreated and treated silicones at static conditions: (**a**) total biofilm mass quantification assessed by crystal violet; (**b**) cell viability within the biofilms and (**c**) fluorescence microscopy images of live (green) and dead (red) bacteria in the biofilms. The green and red fluorescence images are overlaid. Scale bar corresponds to 100 μm. All data are mean values of three independent experiments. Stars represent the statistical differences between the different groups of samples (** *p* < 0.01; *** *p* < 0.001), ns—not significantly different (*p* > 0.05).

The inhibition of *P. aeruginosa* biofilm formation was further assessed at static conditions using crystal violet and bacterial viability assays. The crystal violet method provides quantitative data for the total biomass inhibition, while the cell viability test provides information for the live bacterial cells attached to the material [28,29]. Strong biofilm formation was observed on the pristine silicone after 24 h of incubation with *P. aeruginosa* bacteria. In addition to the strong antibacterial efficiency of Polymyxin B-coated silicones, these were not able to inhibit the total biomass formation or affect the bacterial cell viability on the surface, when compared with the pristine silicone (Figure 4a,b). Cationic polymers such as Polymyxin B interact with the negatively charged bacterial cells inducing cellular lysis on silicone surfaces [30]. The residual bacterial debris block the direct contact of the peptide with the incoming live bacteria and also serve as anchoring points for them to form biofilm structures. Additionally, there are several lines of evidence that the electrostatic interaction between the peptide and bacteria stimulates the intercellular aggregation and formation of clusters on the surface that are insusceptible to bactericides [30] and also promote the growth of biofilm structures [30,31]. From another point of view, pSBMA coating impeded *P. aeruginosa* catheter colonization and total biomass formation by 55% (Figure 4a) but was not able to affect the viability of *P. aeruginosa* surface-attached biofilm cells (Figure 4b). The live bacterial cells may further spread to other parts of the device or living tissues limiting the long-term application of this coating. Importantly, simultaneously deposited pSBMA and Polymyxin B showed higher inhibition of total biomass formation when compared with the pristine silicone and catheters coated individually with pSBMA or Polymyxin B. Up to 97% inhibition of *P. aeruginosa* biofilm and four-log reduction in bacterial cell viability within the biofilm was achieved due to the synergistic action of both antifouling and bactericidal actives (Figure 4a,b). The increase in the silicone hydrophilicity due to the zwitterionic component of the coating decreases the free surface energy and reduces the initial protein adherence and bacterial anchoring [32], allowing the peptide to exert its bactericidal activity.

The biofilm formation and viability of the cells were also assessed using fluorescent microscopy after staining with a Live/Dead Kit (Figure 4c). This assay allows the visualization of dead and alive bacterial cells within the biofilm [33]. The microscopic observations (Figure 4c) were in corroboration with the results from crystal violet and live bacterial cells count assays and further validated the potential of the developed nanoenabled coatings to prevent the *P. aeruginosa* biofilm formation on silicone surfaces. The untreated siliones and silicones coated with Polymyxin B showed very well established *P. aeruginosa* biofilms, and a large amount of green-stained bacterial cells was obtained for the pSBMA coatings, indicating their inefficiency in inhibiting biofilm growth reduction in the biofilm, but most of the cells were live, as obtained in the other tests. In contrast, the adhered *P. aeruginosa* cells on the silicone surface coated with pSBMA@PM nanocomposites were significantly reduced, confirming the strong antifouling and antibacterial features of the developed nanoenabled coating.

### 3.5. Biofilm Inhibition Tests in an In Vitro Model of Catheterized Bladder

**Figure 5:**
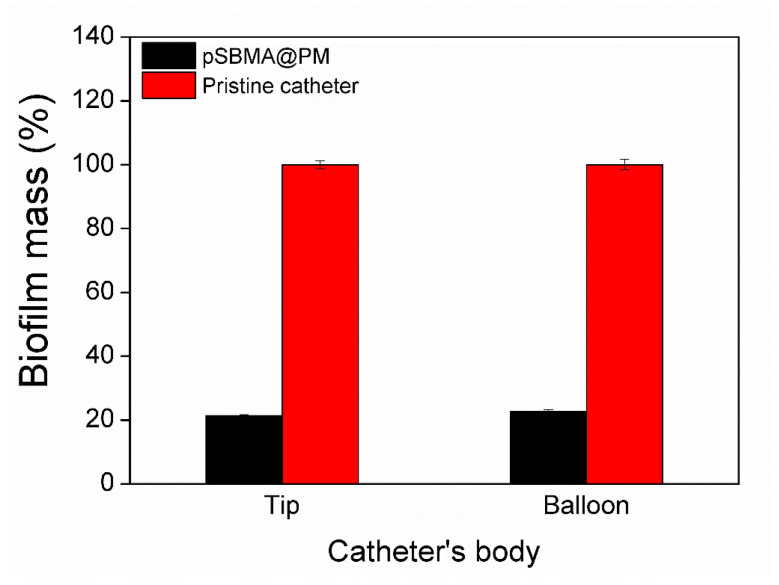
In vitro biofilm inhibition tests in catheterized bladder model with artificial urine recirculation. Total biofilm quantification of *P. aeruginosa* biofilm mass on pSBMA@PM-coated urinary catheters.

pSBMA@PM NPs were produced and coated onto silicone Foley urinary catheters in order to validate the biofilm inhibition activity of the coatings in conditions mimicking the catheter in use. Treated and untreated Foley urinary catheters were inserted in an in vitro model of the human bladder and constantly supplied with artificial urine (1 mL min^−^^1^) during 7 days, the lifetime period for clinical application. After 7 days, the catheters were removed and the formed total biofilm mass on the pristine, and pSBMA@PM catheters was evaluated. The pSBMA@PM coating led to an 80% reduction in *P. aeruginosa* total biofilm formation on the catheter’s tip and balloon due to the bifunctional hybrid nanocomposites (Figure 5). The balloon of the catheter is inflated inside the bladder and is entirely immersed in urine over the time of catheterization. This part of the indwelling urinary catheters is considered the most susceptible to bacterial colonization and the consequent establishment of antibiotic-resistant biofilms by urinary tract pathogens.

### 3.6. Biocompatibility Assessments

**Figure 6:**
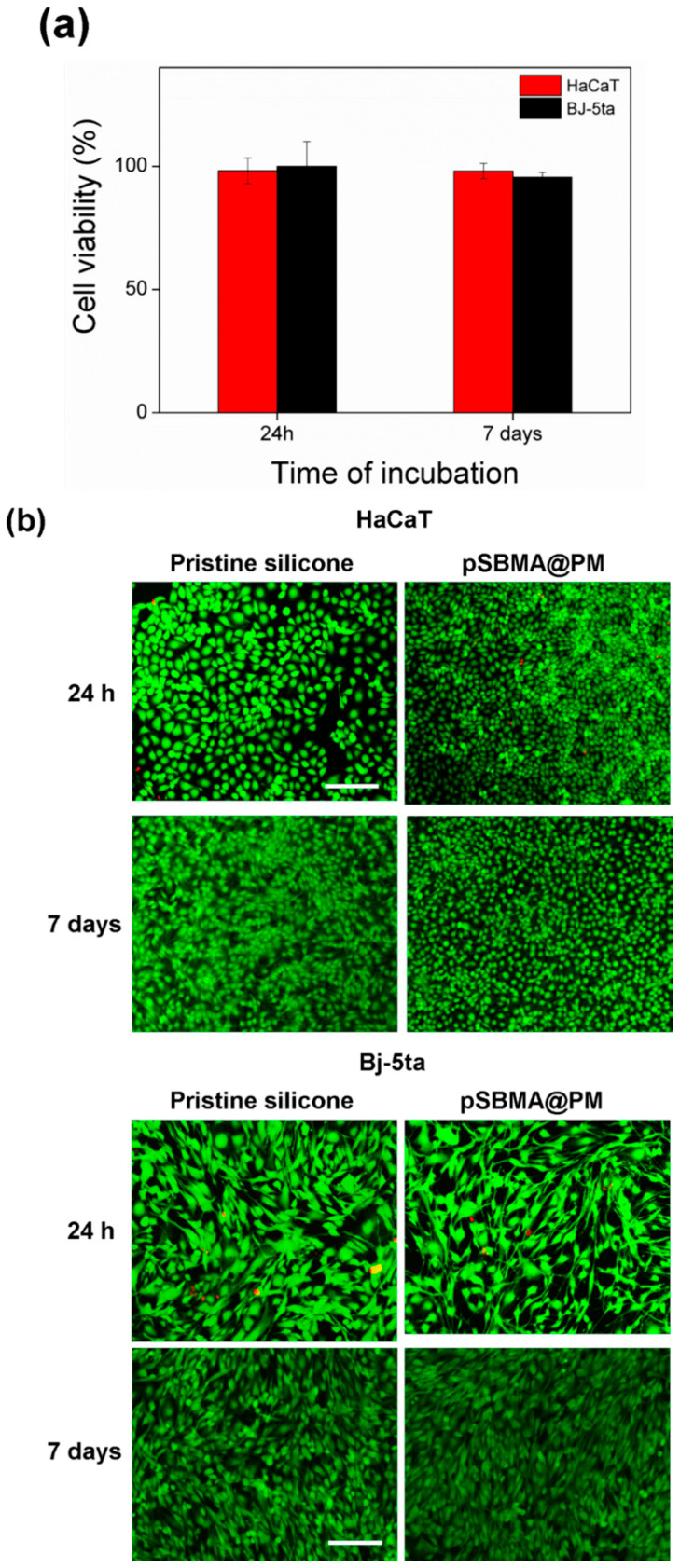
Viability of HaCaT and BJ-5ta cell lines after exposure to the pSBMA@PM-coated silicones assessed by (**a**) AlamarBlue and (**b**) Live/Dead Kit assays. The green and red fluorescence images are overlaid. Scale bar corresponds to 100 μm.

Polymyxin B targets the bacterial cell membranes and in high amounts can induce toxicity to mammalian cells. Nanosized materials, on the other hand, possess unique psychochemical properties and may also induce toxicity. Thus, the evaluation of the toxicity of the engineered coatings is an essential issue for their biomedical application. pSBMA@PM silicone materials were subjected to cytotoxicity tests using two mammalian cell lines—namely, fibroblasts and keratinocytes. The results obtained after the cells’ exposure to the hybrid coating for 24 h and 7 days demonstrated that the pSBMA@PM-coated materials did not affect significantly human cell viability, and more than 95% of the cells were viable (Figure 6a), probably due to the insufficient Polymyxin B amount associated with human cell toxicity. The results from the Live/Dead Kit tests confirmed the biocompatibility of this bifunctional material and did not show changes in the morphologies of mammalian cells (Figure 6b). Considering the excellent biocompatible properties, the developed hybrid coatings could be further evaluated in vivo without considering the possible cytotoxic implication.

## 4. Conclusions

Silicone materials were coated with hybrid zwitterionic/peptide NPs in a one-step environmentally friendly water-based sonochemical process performed at RT, without the need of surface pretreatment or any coatings additive. The engineered nanoenabled coatings showed increased hydrophilicity and reduced protein adsorption, important parameters governing the initial steps of sessile bacterial growth on indwelling medical devices. Moreover, the complementary mode of action of the nanoformulated Polymyxin B and pSBMA resulted in an eight-log reduction in the free-floating *P. aeruginosa* growth and 97% inhibition of resistant biofilm establishment on silicone material. Importantly, the biofilm formation on the SBMA@PM-coated samples was reduced by about 80%, compared with the biofilm produced on the balloon of the pristine catheters, in a dynamic setup simulating the real usage conditions of the device. These coatings did not induce changes in the metabolic activity and morphology of human cells for the same time frame of catheter application, and therefore, the designed dual-targeting approach could be an effective alternative for reducing CAUTIs.

## Data Availability

The data used to support the findings of this study are available from the corresponding author on request.
